# A one-hour walk in nature reduces amygdala activity in women, but not in men

**DOI:** 10.3389/fpsyg.2022.931905

**Published:** 2022-09-27

**Authors:** Sonja Sudimac, Simone Kühn

**Affiliations:** ^1^Lise Meitner Group for Environmental Neuroscience, Max Planck Institute for Human Development, Berlin, Germany; ^2^Max Planck Dahlem Campus of Cognition (MPDCC), Max Planck Institute for Human Development, Berlin, Germany; ^3^Max Planck Institute for Human Development, International Max Planck Research School on the Life Course (LIFE), Lentzeallee 94, Berlin, Germany; ^4^Department of Psychiatry and Psychotherapy, University Medical Center Hamburg-Eppendorf, Hamburg, Germany; ^5^Max Planck UCL Centre for Computational Psychiatry and Ageing Research Berlin, Germany and London, UK, Lentzeallee 94, Berlin, Germany

**Keywords:** environment, nature, urban, sex, brain, amygdala, stress

## Abstract

Urban dwellers are more likely to develop mental disorders such as mood and anxiety disorder as well as schizophrenia compared to rural dwellers. Moreover, it has been demonstrated that even short-term exposure to nature can improve mood and decrease stress, but the underlying neural mechanisms are currently under investigation. In the present intervention study we examined the effects of a one-hour walk in an urban vs. natural environment on activity in the amygdala, a brain region previously associated with stress processing. Before and after the walk 63 participants underwent an fMRI paradigm inducing social stress. Since there is a pronounced gap in the literature regarding interindividual differences in stress-related neural effects of urban and natural environments, we set out to explore sex differences. We observed that amygdala activity decreased after the walk in nature, but only in women, suggesting that women may profit more from salutogenic effects of nature. Moreover, performance on the arithmetic tasks improved in women after the walk in nature, whereas men performed better after the walk in the urban environment. To our knowledge, this is the first study to report differencial tendencies in men and women concerning the stress-related neural activity as an effect of acute exposure to urban vs. natural environments. Furthermore, our findings highlight the importance of sex differences when exploring effects of the environment on brain function and stress. Evidence for beneficial effects of nature on stress-related brain regions may inform urban design policies to focus on providing more accessible green areas in cities and this study suggests that sex differences in experiencing the environment should be taken into consideration.

## Introduction

Although living in an urban environment may have benefits, such as accessibility to education, social services, and amenities (Cohen, 2006), it is likewise linked to increased levels of mental disorders such as mood and anxiety disorders, depression and schizophrenia (Peen et al., 2007). Moreover, it has been repeatedly shown that short visits to nature can be beneficial to cognition and mental health. A growing body of empirical research has demonstrated affective (White et al., 2013; Berto, 2014; Barnes et al., 2019) and cognitive benefits of nature exposure (Berman et al., 2008; Ohly et al., 2016; Stevenson et al., 2018).

The pathways and mechanisms by which nature influences psychological well-being are still unclear. The biophilia hypothesis (Wilson, 1984) states that humans feel an innate affection towards all living beings and that this attitude is rooted in our evolutionary past. Based on this account, Attention Restoration Theory (ART) (Kaplan and Kaplan, 1989) proposes that exposure to natural environments restores voluntary attention. Since nature is filled with intrinsically fascinating stimuli (e.g., trees, lakes), it evokes involuntary attention, allowing voluntary attentional processes to recover. Therefore, after exposure to a natural environment, abilities that depend on voluntary attentional mechanisms, such as working memory, should improve (Berman et al., 2008). In accordance with ART, it has been shown that short-term exposure to nature can restore directed attention and improve cognitive capacity (Berman et al., 2008; Ohly et al., 2016; Stevenson et al., 2018). In contrast, Stress Reduction Theory (SRT) (Ulrich et al., 1991) posits that features found in nature such as vegetation, complexity and absence of threat evoke affective responses that lead to restorative processes. In line with SRT, several studies have demonstrated that exposure to natural environments can improve mood (Berman et al., 2008; Hartig et al., 2014; McMahan and Estes, 2015) and have beneficial effects on stress (Oh et al., 2017; Tost et al., 2019). These results are also supported in studies with physiological indicators of stress, showing that heart rate, blood pressure, and cortisol levels decreased after exposure to natural compared to the urban environments (Park et al., 2010; Lee et al., 2011; Mao et al., 2012; Ochiai et al., 2015; Lanki et al., 2017; Ewert and Chang, 2018).

However, the neural mechanisms behind the effects of nature and urban exposure on stress remain largely unstudied. In cross-sectional studies, a positive association was shown between forest coverage around older adults’ home and amygdala integrity (Kühn et al., 2017), as well as lower amygdala activity during social stress in rural compared to urban dwellers (Lederbogen et al., 2011), indicating that nature in the neighborhood may have salutogenic effects on the amygdala. On the other hand, research investigating the brain-related effects of short-term exposure to natural vs. urban environment has focused on rumination. A study in which participants went on a 90-min walk in an urban vs. a natural environment showed that self-reported rumination as well as activity in the subgenual prefrontal cortex (sgPFC), associated with rumination, decreased only after the walk in nature, suggesting that exposure to nature may be beneficial in reducing rumination and activity in its neural correlates (Bratman et al., 2015).

To examine the causal effects of acute exposure to natural and urban environments on stress-related neural mechanisms, we conducted a functional magnetic resonance imaging (fMRI) intervention study, measuring a change in stress-related brain regions after a one-hour walk in an urban vs. a natural environment. We observed that during the Fearful Faces Task (FFT), activity in the amygdala, a stress-related brain region, decreased after the walk in nature, whereas it remained stable after the walk in an urban environment, indicating that acute exposure to nature may have salutogenic effects on stress-related brain regions (Sudimac et al., 2022).

Social stress (Lederbogen et al., 2013), experiences due to overcrowding (Kennedy et al., 2009), and lack of green areas in cities (Van den Berg et al., 2010) have been proposed as potential causes of disadvantageous effects of urban environments on mental health. Thus, it has been suggested that spending time in green spaces can attenuate social stress present in urban dwellers (Ulrich et al., 1991). In order to examine the neural mechanism underlying social stress after a walk in a natural vs. urban environment, we employed the Montreal Imaging Stress Task (MIST), a paradigm designed to induce social stress. Comparable to the results of the FFT, a differential effect of environment was observed in the MIST, showing that amygdala activity descriptively decreased during the social stress task only after the walk in nature, suggesting that a one-hour exposure to nature was beneficial for amygdala activity during social stress (Sudimac et al., 2022).

However, little is known about the role of interindividual differences in experiencing urban and natural environments, such as sex. It has been previously shown that women are more connected to nature (Zhang et al., 2014; Dean et al., 2018; Rosa et al., 2020) and appreciate nature’s beauty more than men (Zhang et al., 2014). Studies with children have also shown that girls display stronger emotional affinity toward nature than boys (Bagot and Gullone, 2003; Larson et al., 2010). Furthermore, it has been reported that women are more eco-friendly and more concerned about the environment than men (Zelezny et al., 2000). Women consume less carbon and purchase more green products (Zhao et al., 2021) and, as reported in a cross-national study, they show pro-environmental behavior more frequently than men (Hunter et al., 2004). Sex differences were likewise observed in the relation between urban upbringing and brain structure. Namely, gray matter volume in perigenual anterior cingulate cortex (pACC), a key region for regulation of amygdala activity (Pezawas et al., 2005) previously related with urbanicity and stress (Lederbogen et al., 2011), was negatively correlated with years spent in a city during childhood, but only in men (Haddad et al., 2015).

Since sex differences were reported in connectedness to nature (Zhang et al., 2014; Dean et al., 2018; Rosa et al., 2020) and in the association between urban upbringing and gray matter volume (Haddad et al., 2015), in this paper we aimed to explore potential sex differences in amygdala activity change after the walk in the natural vs. urban environment.

Based on the aforementioned literature, we predicted that the walk in nature would have a more beneficial effect for amygdala activity in women compared to men. Furthermore, based on the previous studies showing that exposure to nature is beneficial for attention and cognitive capacity (Berman et al., 2008; Ohly et al., 2016; Stevenson et al., 2018), and taking into account the importance of exploring interindividual differences such as sex when investigating the effects of man-made environments on cognition (Tawil et al., 2021), we predicted that cognitive performance on the MIST would improve after the nature compared to the urban walk and examined potential sex differences.

## Materials and methods

### Participants

The sample consisted of 63 participants (29 females, total mean age = 27.21 years, *SD* = 6.61, age-range = 18–47 years). The participants were pseudo-randomly assigned either to a nature (32 participants) or an urban walk (31 participants), while controlling for equal distribution of men and women. Participants’ age, occupation, education, income and percentage of participants brought up in a city did not significantly differ between the two groups. An overview over the control variables in the two conditions and details about participant recruitment were previously reported elsewhere (Sudimac et al., 2022). Participants were told that they would take part in a magnetic resonance imaging (MRI) study in which they would go for a walk, but were not informed about the research question of the study. All participants were fluent in German, right-handed, and were not diagnosed with any psychological or neurological disorder.

The study was approved by the Local Psychological Ethical Committee at the Center for Psychosocial Medicine at University Medical Center Hamburg-Eppendorf in Hamburg, Germany (LPEK-0054). We obtained written informed consent from all participants and they received monetary compensation.

### Study procedure

**Figure 1:**
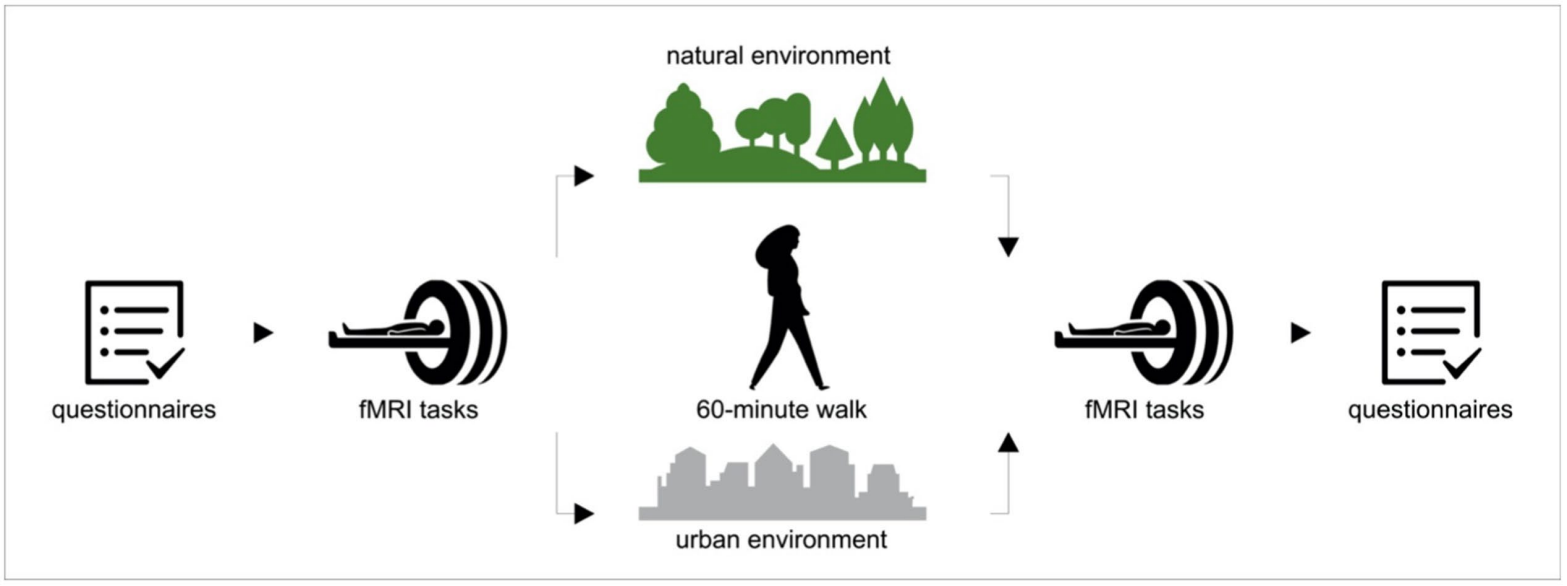
Flowchart of the study procedure. Upon arrival participants filled out the questionnaires and then underwent the fMRI scanning procedure, consisting of Montreal Imaging Stress Task (MIST) and Fearful Faces Task (FFT). Subsequently, participants were randomly assigned to go either for a 60-min walk in either a natural or an un urban environment. After the walk participants again underwent the fMRI procedure and at the end they filled out the questionnaires.

**Figure 2:**
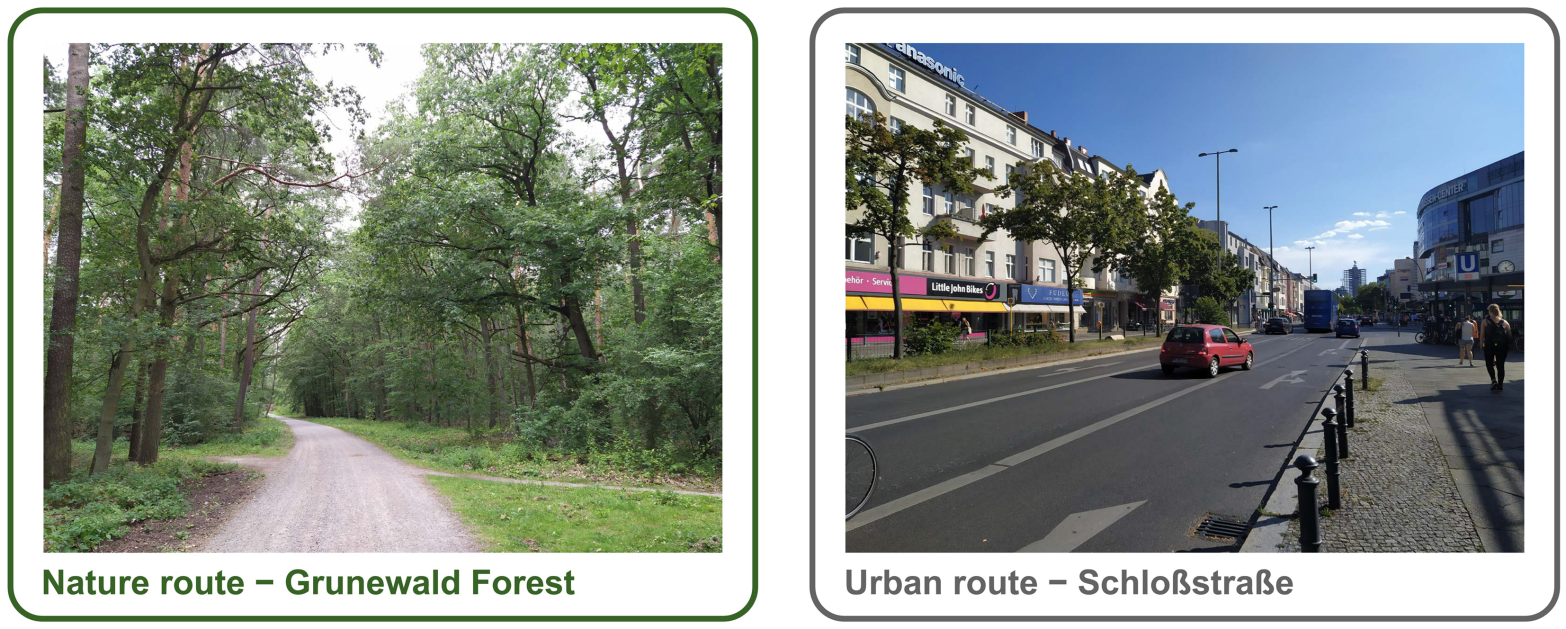
Location of the nature and urban walk. Left: Sample picture of the route in the natural environment in Grunewald forest, the largest green area in the city of Berlin. Right: Sample picture of the walk in the urban environment Schloßstraße, a busy shopping area with traffic in Berlin.

The experiment was conducted in late summer/fall 2019 between 10:00 a.m. and 5:00 p.m. The flowchart of the study procedure is shown on Figure 1. Upon arrival, participants signed the informed consent, filled out the questionnaires and performed a working memory task. Subsequently, the participants underwent an fMRI scanning procedure that included a resting state sequence with questions on rumination (Kühn et al., 2013), the MIST (Dedovic et al., 2005) and the FFT (Mattavelli et al., 2014). After the scanning session, participants were randomly assigned to a 60-min walk in either a natural (Figure 2, left) or urban environment (Figure 2, right). Even though the definition and also the dichotomy of ‘natural’ and ‘urban’ environment has been a subject of debate (Karmanov and Hamel, 2008), the ‘natural environment’ we refer to is an urban forest, the largest green area in the city of Berlin (Grunewald forest; Figure 2, left), whereas ‘urban environment’ refers to a busy street in one of the city centers in Berlin with shopping malls (Schloßstraße; Figure 2, right). As recommended in a recent review (Barnes et al., 2019), we previously reported the exact locations of the walk and the characteristics of the urban and natural environments (Sudimac et al., 2022).

The participants were shown the walk on a map (straight path) and subsequently they were picked up at the lab and brought by taxi to the starting point of the walk. They carried a mobile phone that logged their global positioning system (GPS) data during the walk, to ensure that they walked the intended route. Participants went on the walk alone and were instructed not to enter shops or use their mobile phones, to avoid potential distraction. They were given a bagged lunch that they could eat during the walk. After 30 min, an alarm signal went off on the phone, and participants were instructed to turn around and continued the walk back to the starting point. Here they were picked up by a taxi and brought back to the lab.

After the walk the same fMRI scanning procedure was repepated, with one additional stress-inducing task, the Social-Evaluative Threat task (SET; Wager et al., 2009), a modified version of the Trier Social Stress Test (TSST; Kirschbaum et al., 1993), also meant to induce social stress and administered only after the walk at the end of the MRI session, since we reasoned the cover story would not have been credible twice. Finally, participants filled out the questionnaires and were debriefed and informed about the aim of the study.

### Functional imaging paradigm

The order of the MIST and the FFT was counterbalanced between participants, but their respective order was the same for each participant at pretest and posttest. The tasks were presented via a projector and mirror system and the participants answered using an MR-compatible response box.

#### Montreal Imaging Stress Task

The Montreal Imaging Stress Task (MIST; Dedovic et al., 2005) is a computerized fMRI-adapted paradigm, based on the TSST (Kirschbaum et al., 1993) and was administered in order to induce social stress, since the SRT hypothesizes that nature’s restorative potential is most evident when the individual is stressed (Ulrich et al., 1991). In the MIST participants solve mental arithmetic tasks with a difficulty and time limit designed to be just beyond participant’s cognitive capacities. The MIST consisted of three different conditions: Experimental, Control, and Rest.

**Figure 3:**
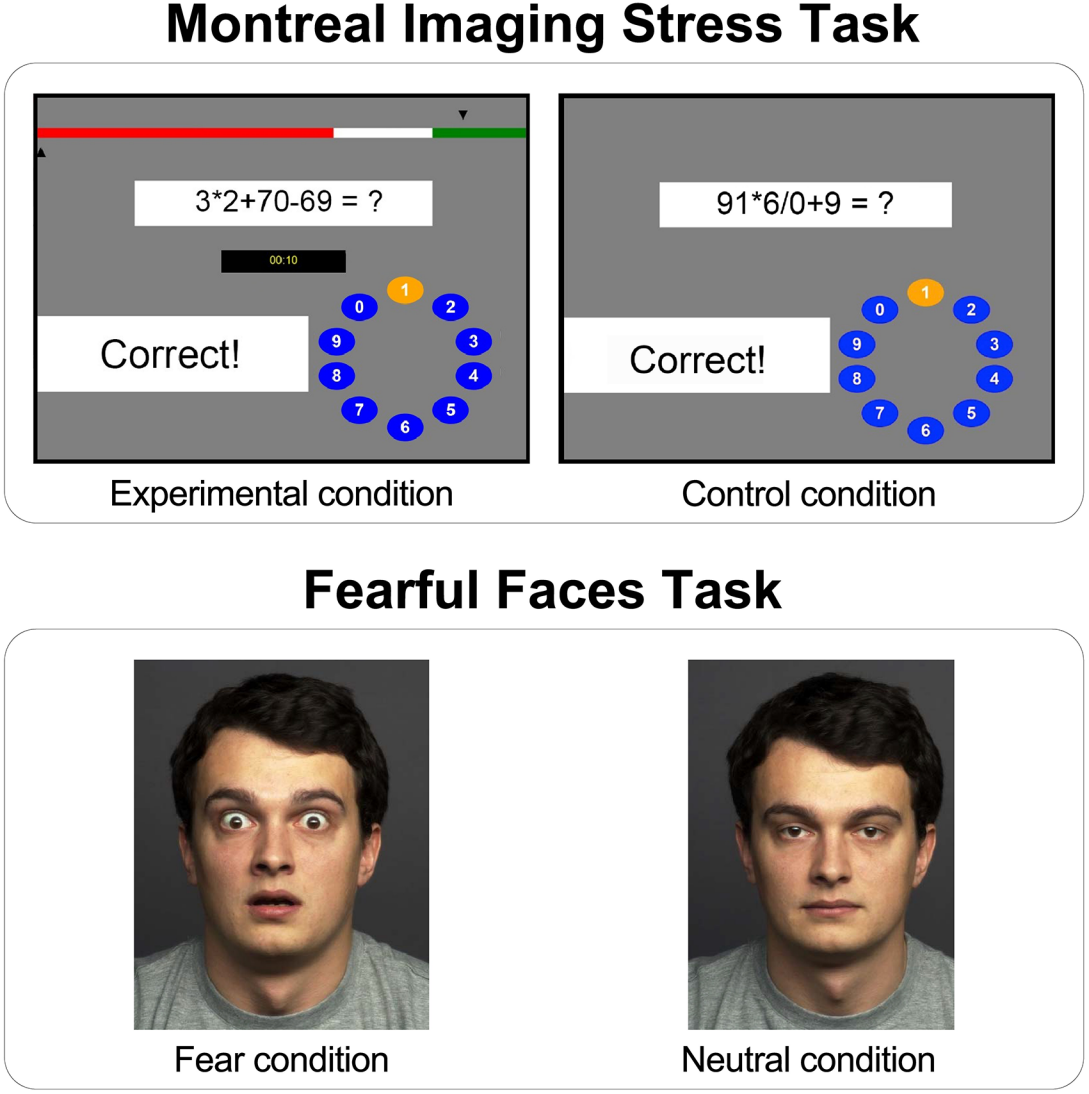
Graphical user interface of the Montreal Imaging Stress Task (MIST) and the Fearful Faces Task (FFT). Above left: Experimental condition within the MIST with a bar presenting participant’s performance (bottom arrow) and fake-average performance (top arrow), the mental arithmetic task, the field showing remaining time for the task, the feedback field and the rotary dial for the response submission; Above right: Control condition within the MIST with the mental arithmetic task, the feedback field and rotary dial; Below left: Example fearful facial expression stimulus within the FFT (Fear condition); Below right: Example neutral facial expression stimulus within the FFT (Neutral condition).

In the beginning of the task there was a training session in which the participant’s ability to perform mental arithmetic was evaluated, without a time limit or progress bar, to calibrate for a default time limit in the Experimental condition. In the Experimental condition the information about individual performance and a fake-average performance of all participants was presented after each response with arrows on a bar above the arithmetic tasks (Figure 3, above left). This fake-average performance was consistently considerably better than the individual’s performance in order to enhance social stress. In the Experimental condition, the MIST program reduced the time limit to 10% less than the participants’s average time after three correctly solved tasks. Conversely, if the participants responded on three consecutive tasks incorrectly, the program increased the time limit for the following tasks by 10%. This staircase procedure in the Experimental condition lead to a range of about 20 to 45% of correct answers (Dedovic et al., 2005). The mathematical arithmetic tasks were designed so that only one digit between 0 and 9 was the correct response. In order to respond, participants selected a digit on the rotary dial from 0 to 9 by pressing the left or the right button on the button box to highlight the neighboring left or right number until they reached the number they intended to respond; in that case the middle button was used to confirm the answer. The participant’s answer was compared with the correct answer for the arithmetic task and the feedback “Correct” or “Incorrect” was shown in the feedback field. If the time for the arithmetic task ran out, the feedback “Time out” was displayed (Dedovic et al., 2005).

In the Control condition the mental arithmetic tasks had the same level of difficulty as in the Experimental condition, but the participant’s performance as well as the fake-average performance of all participants was not displayed and there was no time limit for solving the task (Figure 3, above right). The feedback for each task was also displayed, but since there was no time limit, average correct performance in the Control condition is around 90% (Dedovic et al., 2005).

In the Rest condition, treated as a baseline, the participants saw the rotary dial and empty fields for arithmetic tasks and the feedback, but no task was displayed and the participants were asked to simply look at the screen (Dedovic et al., 2005).

#### Fearful Faces Task

An adapted version of the Fearful Faces Task (FFT; Mattavelli et al., 2014) was used, designed to measure amygdala activity during fearful and neutral facial expressions. While in the MRI scanner, participants were presented with stimuli consisting of 15 male and 15 female faces, each depicting fearful (Fear condition; Figure 3, below left) or neutral facial expression (Neutral condition; Figure 3, below right). Both fearful and neutral facial expressions were shown either for 1,000 ms (unmasked stimuli) or for 17 ms, followed by a mask with neutral facial expressions presented for 983 ms (masked stimuli), since the amygdala has been shown to respond to masked stimuli even when most of the participants were not aware of their presence (Ohman et al., 2007; Kim et al., 2010; Brooks et al., 2012).

We used the set of 60 stimuli from the FACES database by the Max Planck Institute for Human Development in Berlin (Holland et al., 2019), consisting of face photographs on a gray background, matched on size and luminance. The fMRI paradigm consisted of 22 blocks with 6 pictures interleaved with a 200 ms break between pictures. Each block was followed by a white fixation cross presented for 9 s. In order to monitor the participants’ attention, the fixation cross was red on two occasions and participants were instructed to press the button on the response box as soon as they would saw the red cross on the screen. The order of the stimuli was randomized within 10 versions of the FFT, and the task version was introduced in the fMRI data analysis as a covariate. The whole task sequence lasted 8 min and 28 s. The task was presented via a projector and mirror system and the participants answered using the response box. The FFT was presented using the software Presentation (version: 19.0).

### Behavioural data

#### Performance on the Montreal Imaging Stress Task

Performance on the MIST was defined as the percentage of correct answers and mean reaction time (RT) necessary to solve the mental arithmetic tasks in the Experimental condition, during social stress. The percentage of correct answers was analyzed with a two-way ANOVA with environment as a between-subject factor (urban vs. natural) and time as a within-subject factor (before vs. after the walk) using the ezANOVA function from the R package ez (Lawrence and Lawrence, 2016). The same analysis was performed separately within subsamples of women and men. Since the Shapiro–Wilk normality test indicated that female and male subsamples were not normally distributed, we analyzed the percentage of correct answers within each of the subsamples with robust ANOVA using the R package WRS2 (Mair and Wilcox, 2020).

In the RT analysis two participants had a RT that was more than 2.5 SD higher than the mean, therefore they were treated as outliers and excluded from further analysis. The RT was as well analyzed with a two-way ANOVA with environment as a between-subject factor (urban vs. natural) and time as a within-subject factor (before vs. after the walk). Since the RT was not normally distributed across the sample nor across the male subsample, the RT analysis was also performed with robust ANOVA using the R package WRS2 (Mair and Wilcox, 2020). The RT in the female subsample was analyzed with ANOVA using the R package ez (Lawrence and Lawrence, 2016).

*Post-hoc t*-tests were subsequently conducted for both the percentage of correct answers and the RT in order to examine if there were changes in performance after the walk in the urban and in the natural environment within each of the subsamples.

#### Questionnaires

Behavioural measures included questionnaires assessing mood [German version of Positive and Negative Affect Schedule, PANAS (Krohne et al., 1996)], perceived stress during the previous hour [adapted German version of Perceived Stress Scale, PSS (Klein et al., 2016)], rumination during the previous hour [adapted Rumination subscale from German version of Rumination Reflection Questionnaire, RRQ (Elkhaouda, 2010)], and perceived restorativeness [German version of Perceived Restorativeness Scale, PRS (Schönbauer, 2013)], in addition to a computerized Digit Span Backwards (DSB) task, which assesses working memory (Berman et al., 2008).

All behavioural measures were administered before and after the walk, except for the PRS which assesses the perceived restorativeness of an environment and was therefore only administered after the walk. Additionally, participants filled out a sociodemographic questionnaire, reported on the weather during the walk and the overall pleasantness of the walk, and responded to a German version of the Connectedness to Nature questionnaire (Cervinka et al., 2009). The results of the analysis of this data as well as of the physiological data (electrodermal activity and heart rate) were previously reported (Sudimac et al., 2022).

### Magnetic resonance imaging

#### Data acquisition

All images were acquired on a Siemens Tim Trio 3 T scanner (Erlangen, Germany) using a 32-channel head coil. The T1-weighted images were obtained using a three-dimensional T1-weighted magnetization prepared gradient-echo sequence (MPRAGE; repetition time (TR) = 2,500 ms; echo time (TE) = 4.77 ms; TI = 1,100 ms, acquisition matrix = 256 × 256 × 192, flip angle = 7°; 1x1x1 mm^3^ voxel size). Whole brain functional images were collected using a T2*-weighted echo-planar imaging (EPI) sequence sensitive to BOLD contrast (TR = 2000 ms, TE = 30 ms, acquisition matrix = 216 × 216 × 129, flip angle = 80°, slice thickness = 3.0 mm, distance factor = 20%, FOV = 216 mm, 3 × 3 × 3 mm^3^ voxel size, 36 axial slices, using GRAPPA).

#### Data preprocessing

Functional imaging data were preprocessed and analyzed using Statistical Parametric Mapping software (SPM12^1^). EPIs were corrected for slice timing and head motion and transformed into the stereotactic normalized standard space of the Montreal Neuroimaging Institute (MNI) using the unified segmentation algorithm. Finally, spatial smoothing with a 6-mm full width at half-maximum (FWHM) Gaussian kernel was performed. The voxel size was not changed during preprocessing but kept in the original acquisition dimension (3 × 3 × 3 mm^3^).

#### Data analysis

##### Montreal Imaging Stress Task

At the first level analysis estimates of functional activation during conditions (Experimental, Control and Rest) were obtained using a blocked analysis. A high-pass filter (cut-off 520 s) was applied. We then used an approach based on our region of interest (ROI), bilateral amygdala. The bilateral amygdala mask was derived from the Automated Anatomic Labelling atlas 2 (Rolls et al., 2015) and had a volume of 3,744 mm^3^.

We extracted the beta values for each of the contrasts (Experimental > Rest and Control > Rest) within the bilateral amygdala, using the marsbar toolbox [version 0.44 (Brett et al., 2002)]. As previously reported, we observed a significant interaction in the MIST between environment and time in amygdala pooled activity of the Experimental and Control condition, which descriptively decreased after the walk in nature and remained stable after the walk in the urban environment (Sudimac et al., 2022). In the present study we examined the change in amygdala activity in the MIST in male and female subsample separately. In both subsamples a two-way ANOVA was conducted with environment as a between-subject factor (urban vs. natural) and time as a within-subject factor (before vs. after the walk) in amygdala pooled activity of Experimental and Control condition. Subsequently, two-tailed *post-hoc t*-tests were conducted in order to examine if the environment-by-time interaction was driven by a change in amygdala activity after the walk in the urban or in the natural environment.

##### Fearful Faces Task

At the first level analysis of the FFT, estimates of functional activation during each condition (unmasked Fear, unmasked Neutral, masked Fear, masked Neutral, Response) were modelled using an event-related paradigm. A high-pass filter (cut-off 128 s) was applied and the ROI-based approach was used, focusing on the bilateral amygdala.

We reasoned that the intervention, namely a one-hour walk, would globally affect the stress levels and therewith stress-related brain activity, not only when contrasting the Fear > Neutral condition. Therefore, we examined amygdala activity in the Fear and in the Neutral condition separately, by extracting the blood-oxygen-level dependent (BOLD) signal within the bilateral amygdala using the marsbar toolbox [version 0.44 (Brett et al., 2002)]. We averaged data from unmasked and masked stimuli, because the results were similar.

Within both the male and female subsamples we conducted a two-way ANOVA with environment as a between-subject factor (urban vs. natural) and time as a within-subject factor (before vs. after the walk) in amygdala activity pooled from the Fear and Neutral conditions. Two-tailed *post-hoc t*-tests were performed within the urban and the natural environment to examine if the environment-by-time interaction was driven by a change in amygdala activity after the urban walk or the walk in nature.

## Results

**Figure 4:**
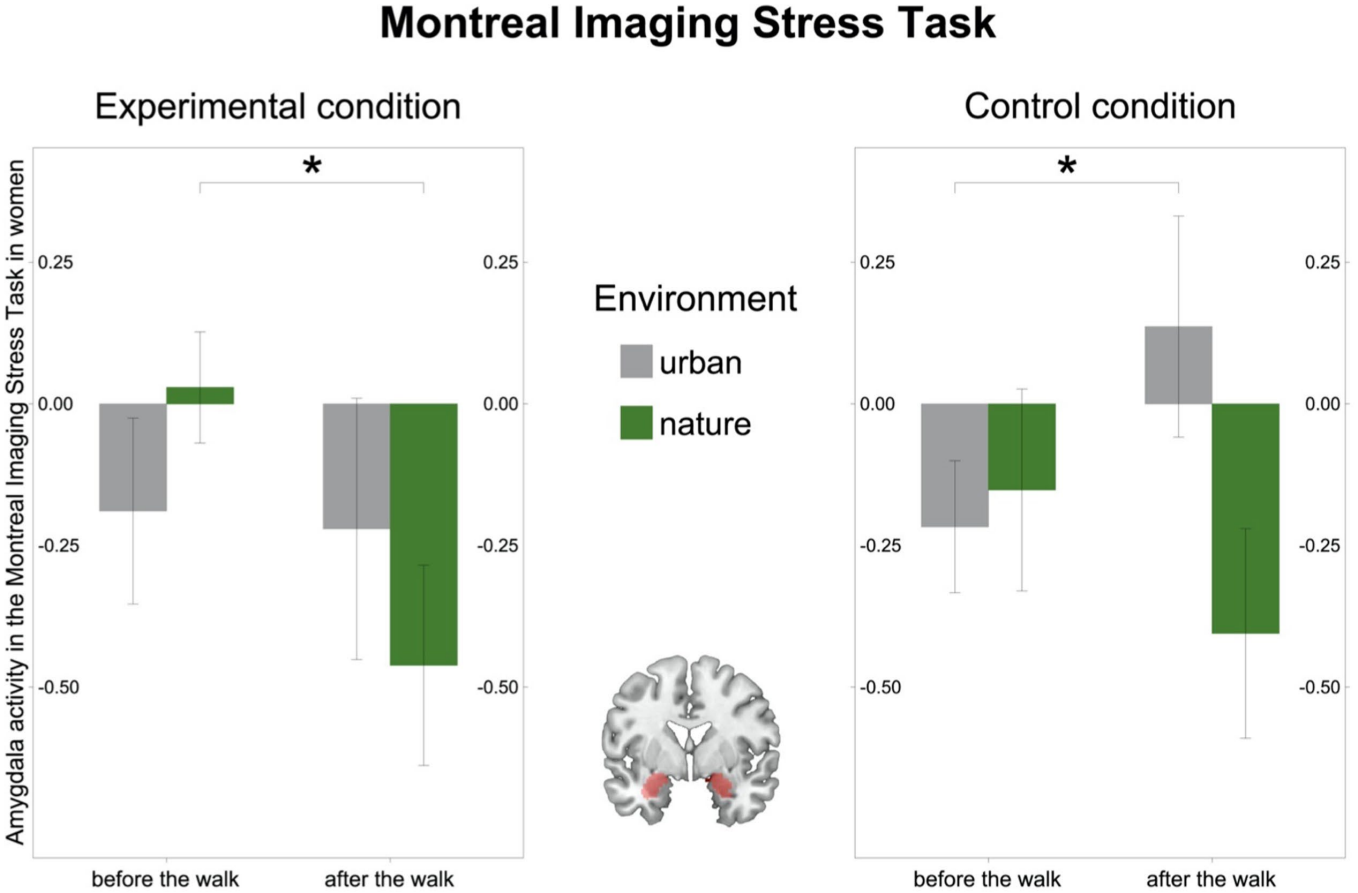
Amygdala activity in the Montreal Imaging Stress Task (MIST) in women. Left: Amygdala activity (beta values) in the Experimental condition in the MIST decreased after the walk in the natural environment in women. Right: Amygdala activity (beta values) in the Control condition in the MIST increased after the walk in the urban environment in women. Middle: Region of interest, the bilateral amygdala as defined in Automated Anatomic Labelling Atlas 2. Significant differences are indicated with asterisks (*p < 0.05); error bars represent one standard error of the mean.

In the MIST, as hypothesized, we observed a significant environment-by-time interaction in the female subsample in pooled amygdala activity from the Experimental and Control conditions [(*F*(27) = 4.74, *p* = 0.038, *η*^2^*_g_* = 0.04); Figure 4]. The interaction was driven by amygdala activity which descriptively decreased after the walk in nature [*t*(14) = 1.89, *p* = 0.080], whereas it remained stable after the walk in the urban environment [*t*(13) = −1.15, *p* = 0.270; Supplementary Tables 1, 2]. Furthermore, the environment-by-time interaction in the female subsample was driven by a decrease in amygdala activity in the Experimental condition after the walk in nature [(*t*(14) = 2.35, *p* = 0.034); Figure 4, left] and by an increase in activation in the Control condition after the walk in the urban environment [(*t*(13) = −2.62, *p* = 0.021); Figure 4, right]. In the male subsample, however, there was no significant environment-by-time interaction in amygdala activity [(*F*(32) = 1.28, *p* = 0.266, *η*^2^*_g_* = 0.008); Supplementary Figure 1] and it remained stable after both the walk in the natural [(*t*(16) = 0.93, *p* = 0.367)] and the urban environment [(*t*(16) = −0.66, *p* = 0.516); Supplementary Tables 1, 2].

**Figure 5:**
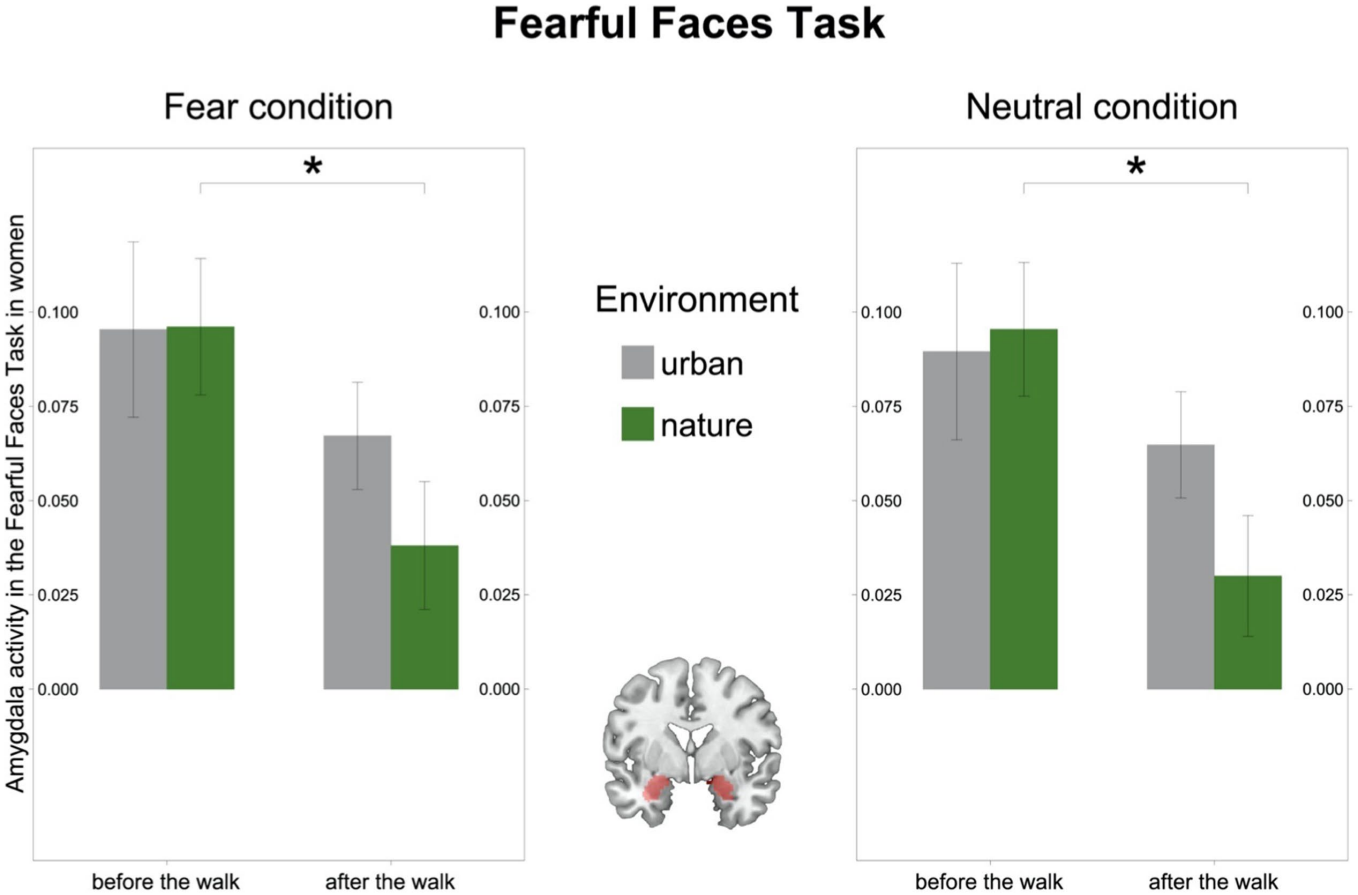
Amygdala activity in the Fearful Faces Task (FFT) in women. Left: Amygdala activity (BOLD signal) in the Fear condition in the FFT decreased after the walk in the natural environment in women. Right: Amygdala activity (BOLD signal) in the Neutral condition in the FFT decreased after the walk in the natural environment in women. Middle: Region of interest, the bilateral amygdala as defined in Automated Anatomic Labelling Atlas 2. Significant differences are indicated with asterisks (*p < 0.05); error bars represent one standard error of the mean.

In the FFT we observed a significant environment-by-time interaction in the male subsample [*F*(32) = 4.37, *p* = 0.045, *η*^2^*_g_* = 0.05; Supplementary Figure 2], driven by an increase in amygdala activity after the walk in the urban environment [(*t*(16) = −1.99, *p* = 0.063)], while there was no significant interaction in the female subsample [*F*(27) = 1.71, *p* = 0.203, *η*^2^*_g_* = 0.02]. However, in line with the results on the MIST, amygdala activity after the walk in nature significantly decreased in women [(*t*(14) = 2.89, *p* = 0.012); Figure 5], whereas it remained stable in men [(*t*(16) = 1.06, *p* = 0.303); Supplementary Tables 1, 2].

Exploratorily, we examined the relationship between the change in amygdala activity after the walk and connectedness to nature score. We observed a positive correlation between the change in amygdala activity on the MIST after the urban walk in women and their connectedness to nature [*r*(12) = 0.55, *p* = 0.043; Supplementary Figure 3], whereas no correlation was found in men [*r*(15) = 0.12, *p* = 0.641].

Regarding the participants’ cognitive performance, defined as percentage of correct answers and mean RT in the Experimental condition in the MIST, we observed no significant environment-by-time interaction neither on the whole sample (Supplementary Table 3), nor when splitting the sample into males and females (Supplementary Table 4). Interestingly, the results revealed that men had a higher percentage of correct answers in the Experimental condition in the MIST [*t*(54.5) = −2.18, *p* = 0.034], compared to women.

*Post-hoc t*-test showed that the percentage of correct answers in the Experimental condition decreased in women after both the urban [*t*(13) = 3.03, *p* = 0.010] and nature walk [*t*(14) = 2.68, *p* = 0.019], as well as in men after the walk in nature [*t*(16) = 2.63, *p* = 0.018], whereas the percentage of correct answers in men did not change after the walk in the urban environment [*t*(16) = 1.51, *p* = 0.151].

**Figure 6:**
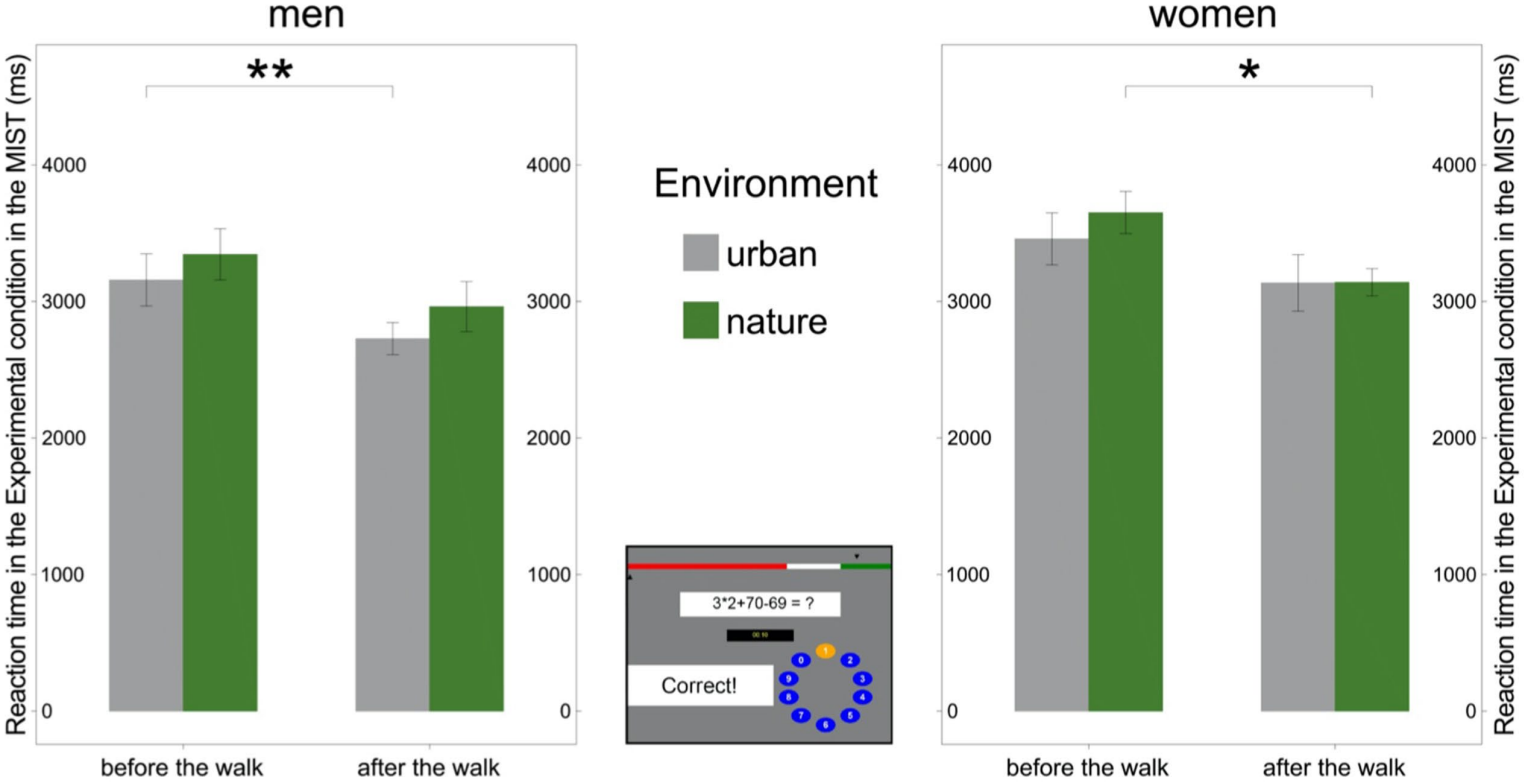
Reaction time in the Experimental condition of the Montreal Imaging Stress Task (MIST) by sex. Left: Reaction time in the Experimental condition of the MIST decreased after the walk in the urban environment in men. Right: Reaction time in the Experimental condition in the MIST decreased after the walk in the natural environment in women. Middle: Experimental condition in the MIST. Significant differences are indicated with asterisks (*p < 0.05; **p < 0.01); error bars represent one standard error of the mean.

However, the mean RT necessary to solve mental arithmetic tasks in the Experimental condition decreased in women only after the walk in nature [*t*(13) = 2.60, *p* = 0.022] and in men only after the walk in the urban environment [*t*(16) = 3.14, *p* = 0.006; Figure 6; Supplementary Table 5]. On the other hand, the RT did not significantly change for women after the walk in the urban environment [*t*(13) = 1.96, *p* = 0.072] nor for men after the walk in the natural environment [*t*(15) = 1.76, *p* = 0.099; Figure 6; Supplementary Table 5].

## Discussion

In order to investigate the causal effects of the environment on stress-related brain regions, we conducted an fMRI intervention study in which a change in the amygdala was measured as an effect of a one-hour walk in an urban vs. natural environment. We have previously shown that activity in the amygdala decreased as the result of a one-hour walk in nature, whereas it remained stable after a walk in the urban environment while participants underwent the FFT. Consistent with this finding, a significant environment-by-time interaction during the MIST indicated that amygdala acvitivity descriptively decreased as well on the social stress task after the walk in the natural environment, whereas it remained stable after the walk in the urban environment, suggesting salutogenic effects of nature exposure on the amygdala (Sudimac et al., 2022).

However, there is a pronounced gap in the literature regarding the role of interindividual differences in the effect of exposure to urban and natural environments on brain regions related to social stress. Since sex differences have been previously reported in connectedness to nature (Zhang et al., 2014; Dean et al., 2018; Rosa et al., 2020), as well as in the association between urban upbringing and gray matter volume (Haddad et al., 2015), here we set out to explore sex differences in amygdala activity change as an effect of the urban vs. nature walk.

In line with our hypothesis, in the MIST in the female subsample we observed an interaction between environment and time, showing that amygdala activity during the MIST descriptively decreased after the walk in the natural environment, whereas it remained stable after the walk in the urban environment. On the other hand, the amygdala activity during the MIST in the male subsample did not significantly change after both the walk in the urban nor in the natural environment. Similar as in the MIST, within the FFT paradigm we observed a decrease in amygdala activity in women after the walk in nature, whereas it remained stable in men. Therefore, these results altogether suggest that the beneficial effects of nature on stress-related brain regions are more pronounced in women.

Interestingly, in the exploratory analysis we observed that the stronger connectedness to nature was in women, the change in their amygdala activity on the MIST after the city walk was higher, whereas no such association was found in men. Namely, the urban walk increased the stress-related neural activity during the social stress task more in women who are strongly connected to nature, than in those who feel less connected.

Moreover, the reaction time in the mental arithmetic task decreased only in the female subsample after the walk in nature, indicating that the natural environment was also more beneficial to cognition in women than in men. On the other hand, the reaction time decreased only in the male subsample after the urban walk, indicating that the walk in the urban environment was beneficial to cognition in men, but not in women. Interestingly, ART posits that nature restores attention, leading to better cognitive performance after nature exposure (Berto, 2014), however our results suggest that this effect is only observed in women, whereas men benefit cognitively from exposure to an urban environment. However, it should be highlighted that the cognitive performance was measured during the social stress task and therefore it might not be comparable with performance in a common working memory task within the ART approach (Ohly et al., 2016). On the other hand, the results showing a decrease in stress-related neural activity after the walk in nature in women are in accordance with SRT, which predicts that exposure to nature leads to recovery from stress.

To the best of our knowledge this is the first study to demonstrate differential tendencies in amygdala activity change in men and women as a causal effect of acute exposure to a natural vs. urban environment. The findings are in line with results from a pilot study showing that after 20-min exposure to an urban forest (Beil and Hanes, 2013), women reported greater decreases in stress than men. Furthermore, the difference in sex in relatedness to nature was also demonstrated in children, showing that girls reported a more intense positive affect related to natural stimuli (Bagot and Gullone, 2003; Larson et al., 2010), as well as a higher frequency of exposure to natural stimuli, whereas boys reported more intense positive affect related to non-natural stimuli (Bagot and Gullone, 2003). Given that women feel more connected nature (Zhang et al., 2014; Dean et al., 2018; Rosa et al., 2020), appreciate more its beauty more (Zhang et al., 2014) and care about the environment more than men (Zelezny et al., 2000), it might be that nature’s relieving effect during social stress is therefore more pronounced in women.

Interestingly, in a previous study in which participants watched 3-D videos with different tree cover density, the relationship between stress recovery and tree densities showed an inverted U-shape in men. Namely, recovery from stress (measured by physiological stress indicators) was positively associated with tree cover density, up to 24%. From 24 to 34% of tree cover density there was no change in stress recovery, whereas higher tree cover density was associated with slower recovery from stress (Jiang et al., 2014). The authors speculate that the slower stress recovery related to high tree density might have been associated with limited sky view and reduction in openness of space, present at higher tree densities. A previous study also reported that natural environments with low openness of space can increase levels of stress (Gatersleben and Andrews, 2013). Since in our study the path where the nature walk took place was mostly surrounded by high coniferous trees, the openness of space was low and the sky view was limited, which, as proposed in the aforementioned study, may have obstructed stress recovery in men after the walk in nature.

To date, few studies have shown that exposure to natural environments had a beneficial effect on cognition in women, whereas the exposure to the urban environment had a beneficial effect on cognition in men. On the other hand, these results are consistent with those of a previous study with children which showed a positive association between nature views from home and benefits in concentration and delayed gratification for girls, but not for boys (Taylor et al., 2002). In correspondence with our finding, it has been shown that stress impairs women’s cognitive capacity, whereas it improves cognitive capacity in men (Schoofs et al., 2013) and leads to hyperarousal in women, but not in men (Bangasser et al., 2018). Given that arousal and cognitive performance have an inverted U-shaped relationship, with optimal arousal leading to optimal performance (Fisk et al., 2018), we speculate that the walk in nature decreased high arousal in women, leading to their better cognitive performance. In the same manner, since men under stress are less aroused than women (Bangasser et al., 2018) and stress enhances cognitive capacity in males (Schoofs et al., 2013), the walk in the urban environment, characterized by high social stress, could have increased their arousal and subsequently improved their cognitive performance. However, we have not explicitly tested for self-reported arousal levels after the stress-inducing fMRI paradigm and therefore we have no data on whether arousal levels differ between men and women before the walk. Thus, we recommend that future studies evaluate self-reported stress and arousal after the stress-inducing paradigm before the walk and directly after the walk.

There may also be additional limitations of the present study. The first limitation is a sample bias, since our sample consisted of young adults, mostly students from Germany, a WEIRD (Western, Educated, Industrialized, Rich, Democratic) country. Future studies should include participants from different age ranges, professions and cultures in order to overcome this bias and, especially, to examine the effect of interindividual differences, such as age, culture, personality traits etc. in experiencing urban and natural environments and in their influence on stress-related brain regions. Secondly, even though the MIST has been widely used to induce social stress in neuroimaging studies (Dedovic et al., 2009), social stress is induced by solving arithmetic tasks under time pressure, and therefore level of induced stress may depend on participants’ mathematical skills. Considering that during social stress male participants performed better than female participants on the MIST and that a wide range of sociocultural factors contribute to sex differences in interests and achievements in mathematics (Halpern et al., 2007), we recommend that future studies that may choose to focus on sex differences should employ a social stress task that does not rely on participants’ previous skills. Thirdly, as discussed in the previous paper (Sudimac et al., 2022), questionnaires given at pretest, before the walk, and at posttest, after the fMRI stress-inducing paradigm, that evaluated the affective state of the participants did not capture the effect of the walk itself, but rather the effect of the stress-inducing paradigm in the scanner and therefore were not used in the analysis with the fMRI data. Finally, it cannot be concluded which features of the nature exposure elicited the decrease in amygdala activity and the reaction time in women, as well as which aspects of the urban environment triggered the decrease in reaction time in men. We would therefore recommend that future studies differentially investigate distinct aspects of natural and urban environments, such as openness of the view, green color, number of people encountered in the environment, noise, odors etc. in order to disentangle specific features of these environments that impact stress-related brain regions.

To summarize, since there is a pronounced gap in the literature regarding interindividual differences in neural effects of exposure to natural vs. urban environments, we examined sex differences. We did so using a social stress paradigm and a fearful faces paradigm focused on activity in the amygdala, a stress-related brain region, as an effect of a one-hour walk in an urban vs. natural environment. We found that amygdala activity decreased after the walk in nature, but only in women, suggesting that women may profit more from the beneficial effects of nature. We also observed that the natural environment improved cognitive performance in women, whereas in men the cognitive performance improved after the walk in the urban environment.

This study is one of the first studies examining stress-related neural effects of an acute exposure to urban vs. natural environments. It is also a seminal study to examine interindividual differences in these effects, such as sex, and to report differential tendencies in men and women regarding their cognitive performance and stress-related neural correlates as a consequence of a short-term exposure to urban vs. natural environments. Therefore, we advise future research not to assume the same salutogenic effects for different population subgroups and to take participants’ sex into account, as well as potentially other interindividual differences when investigating the effect of urban and natural environments on cognition, stress and underlying neural mechanisms. The results of this study are significant beacuse they suggest that natural and urban environments may affect men and women differently, which should be taken in account when designing urban green spaces in a way that would be optimal for citizens’ mental health.

## Data availability statement

The datasets presented in this study can be found in online repositories. The names of the repository/repositories and accession number(s) can be found at: https://osf.io/jazqm/.

## Ethics statement

The studies involving human participants were reviewed and approved by Local Psychological Ethical Committee at the Center for Psychosocial Medicine at University Medical Center Hamburg-Eppendorf in Hamburg, Germany. The patients/participants provided their written informed consent to participate in this study.

## Author contributions

SS designed and coordinated the study, collected the data, performed data analysis, interpreted the results, and wrote the paper. SK had the idea for the study, designed and coordinated the study, supervised data acquisition and data analyses, interpreted the results, and reviewed the manuscript. All authors contributed to the article and approved the submitted version.

## Conflict of interest

The authors declare that the research was conducted in the absence of any commercial or financial relationships that could be construed as a potential conflict of interest.

## Publisher’s note

All claims expressed in this article are solely those of the authors and do not necessarily represent those of their affiliated organizations, or those of the publisher, the editors and the reviewers. Any product that may be evaluated in this article, or claim that may be made by its manufacturer, is not guaranteed or endorsed by the publisher.

## Footnotes

^1^ https://www.fil.ion.ucl.ac.uk/spm/software/spm12/
