# Supplementary material for: A one-hour walk in nature reduces amygdala activity in women, but not in men
Source: Front Psychol. 2022 Sep 27;13:931905. doi: 10.3389/fpsyg.2022.931905 (PMC9556704; doi:10.3389/fpsyg.2022.931905)
Supplement: Supplementary file 1 [file Data_Sheet_1.pdf]

Supplementary Material

1 Supplementary Figures

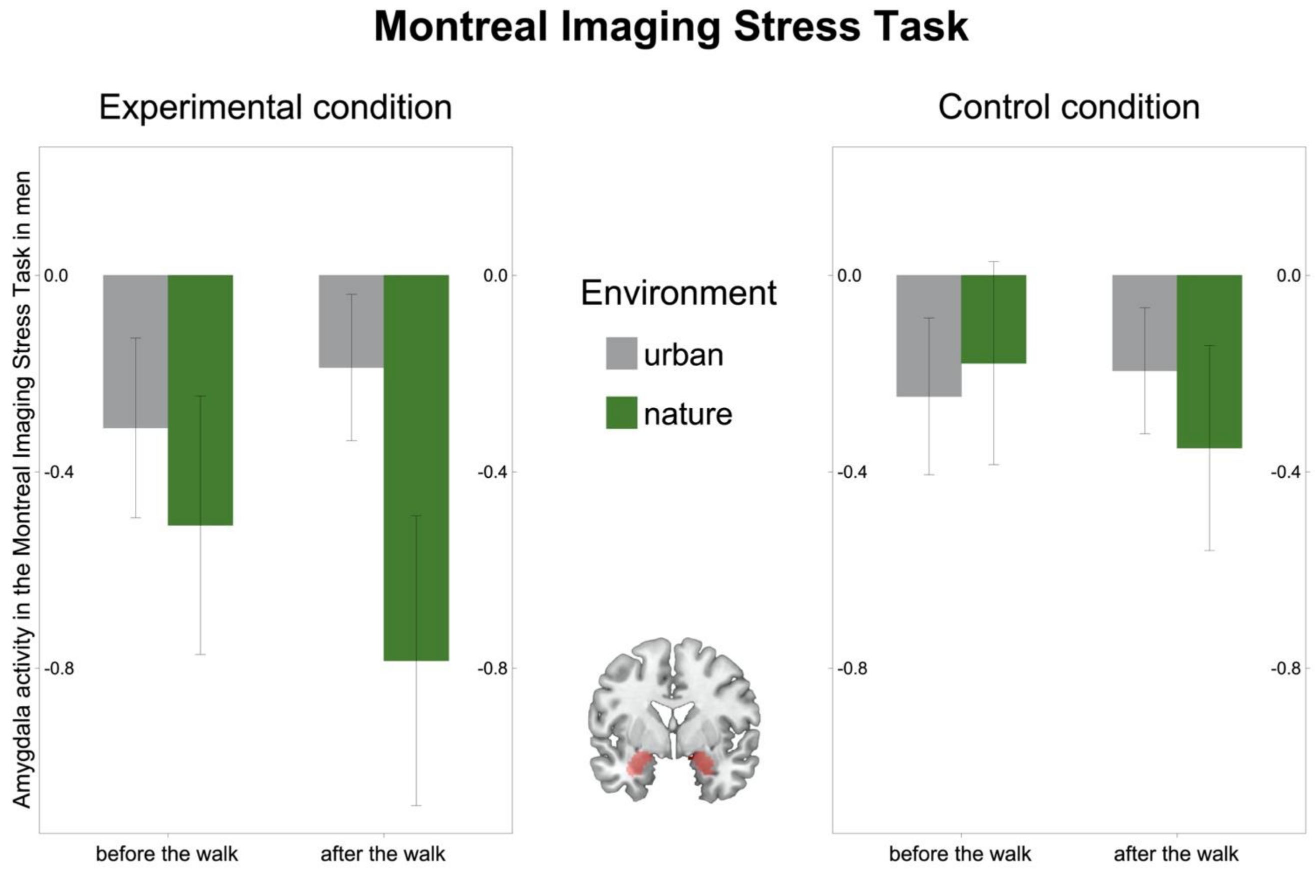

Supplementary Figure 1. Amygdala activity in the Experimental and in the Control condition in the Montreal Imaging Stress Task (MIST) in men. Left: Amygdala activity (beta values) in the Experimental condition in the MIST remains stable after the walk in both urban and natural environment in men. **Right:** Amygdala activity in the Control condition in the MIST remains stable after the walk in both urban and natural environment in men. **Middle:** Region of interest, the bilateral amygdala as defined in Automated Anatomic Labelling Atlas 2.

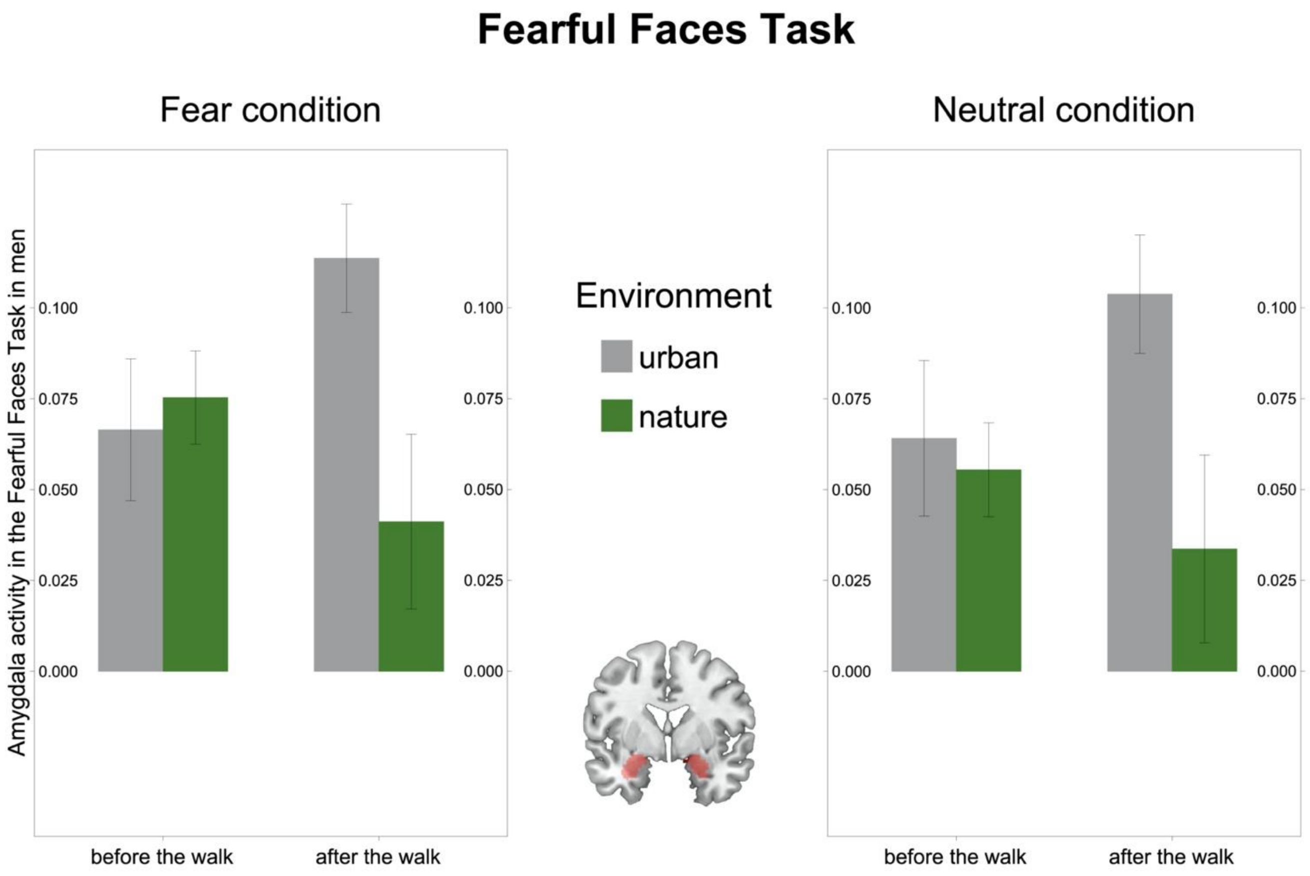

Supplementary Figure 2. Amygdala activity in the Fear and in the Neutral condition in the Fearful Faces Task (FFT) in men. Left: Amygdala activity (BOLD signal) in the Fear condition in the FFT remains stable after the walk in both urban and natural environment in men. **Right:** Amygdala activity (BOLD signal) in the Neutral condition in the FFT remains stable after the walk in both urban and natural environment in men. **Middle:** Region of interest, the bilateral amygdala as defined in Automated Anatomic Labelling Atlas 2.

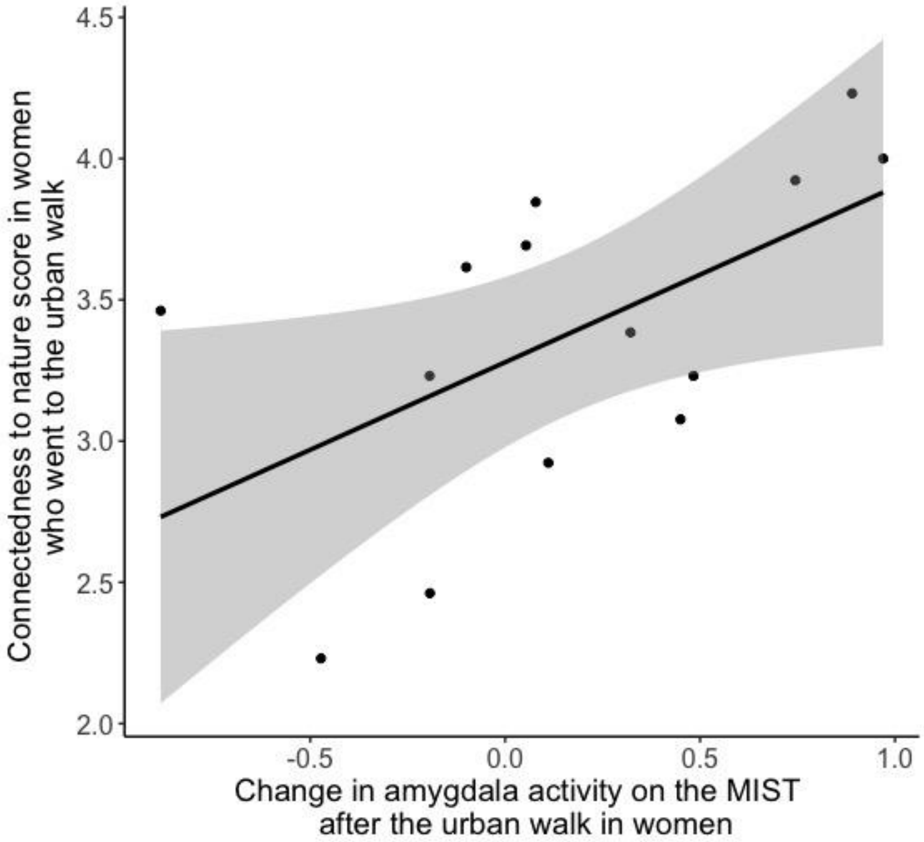

Supplementary Figure 3. Relationship between a change in amygdala activity on the Montreal Imaging Stress Task (MIST) after the urban walk in women and their connectedness to nature.

2 Supplementary Tables

Supplementary Table 1: Post hoc t-test between pretest and posttest for bilateral amygdala activity in the Fearful Faces Task (FFT) and in the Montreal Imaging Stress Task (MIST) by sex.

	**Men**						**Women**					
	**Urban (*n* = 17)**			**Nature (*n* = 17)**			**Urban (*n* = 14)**			**Nature (*n* = 15)**		
**Task**	**t-test**	***df***	***P value***	**t-test**	***df***	***P* value**	**t-test**	***df***	***P value***	**t-test**	***df***	***P* value**
**FFT**	-1.99	16	0.063	1.06	16	0.303	1.65	13	0.122	2.89	14	0.012*
**MIST**	-0.66	16	0.516	0.93	16	0.367	-1.15	13	0.270	1.89	14	0.080

Note: Post-hoc t-tests (pretest vs. posttest) for bilateral amygdala activity in the Fearful Faces Task (pooled activity during Fear and Neutral condition) and in the Montreal Imaging Stress Task (pooled activity during Experimental and Control condition) by sex; *df* = degrees of freedom; $*P < 0.05$, refers to BOLD activity/beta values.

Supplementary Table 2: Descriptive statistics for bilateral amygdala activity in the Fearful Faces Task (FFT) and in the Montreal Imaging Stress Task (MIST) by sex.

	Mean ± *SD*							
	Men				Women			
	Urban (*n* = 17)		Nature (*n* = 17)		Urban (*n* = 14)		Nature (*n* = 15)	
**Task**	**pretest**	**posttest**	**pretest**	**posttest**	**pretest**	**posttest**	**pretest**	**posttest**
**FFT**	0.06±0.07	0.10±0.06	0.06±0.05	0.03±0.09	0.09±0.09	0.07±0.05	0.10±0.07	0.03±0.06
**MIST**	-0.28±0.61	-0.19±0.52	-0.34±0.91	-0.57±0.81	-0.20±0.46	-0.04±0.75	-0.06±0.50	-0.43±0.63

Note: Descriptive statistics for bilateral amygdala activity in the Fearful Faces Task (pooled activity during Fear and Neutral condition) and in the Montreal Imaging Stress Task (pooled activity during Experimental and Control condition) by sex; *SD* = standard deviation, refers to BOLD activity/beta values.

Supplementary Table 3: ANOVA results of the cognitive performance in the Experimental condition of the Montreal Imaging Stress Task (MIST)

**Cognitive performance indicator**	***df***	***F***	***P value***
Percentage of correct answers	61	0.05	0.831
Reaction time	35.98	0.38	0.541

Note: Two-way mixed ANOVA interaction effect of factors environment (urban vs. natural) and time (pretest vs. posttest) on percentage of correct answers and reaction time in the Experimental condition of the Montreal Imaging Stress Task (MIST); Due to non-normal distribution, analysis of the Reaction time was performed using robust ANOVA; *df* = degrees of freedom.

Supplementary Table 4: ANOVA results of the cognitive performance in the Experimental condition of the Montreal Imaging Stress Task (MIST) by sex

	Men				Women			
Cognitive performance indicator	*df*	*F*	*P value*	η^2_g	*df*	*F*	*P value*	η^2_g
Percentage of correct answers	16.56	1.94	0.182	0.02	16.48	1.87	0.190	0.007
Reaction time	19.07	0.48	0.497	0.0002	26	0.50	0.484	0.006

Note: Two-way mixed ANOVA interaction effect of factors environment (urban vs. natural) and time (pretest vs. posttest) on percentage of correct answers and reaction time in the Experimental condition of the Montreal Imaging Stress Task (MIST) by sex; Due to non-normal distribution, analysis of the variables Percentage of correct answers in male and female subsamples and the Reaction time in the male subsample was performed using robust ANOVA; *df* = degrees of freedom. η^2_g= generalized eta-squared effect size.

Supplementary Table 5: Descriptive statistics for mean reaction time for solving mental arithmetic tasks in the Experimental condition in the Montreal Imaging Stress Task (MIST) by sex

**Mean ± *SD***							
**Men**				**Women**			
**Urban (*n* = 17)**		**Nature (*n* = 16)**		**Urban (*n* = 14)**		**Nature (*n* = 14)**	
**pretest**	**posttest**	**pretest**	**posttest**	**pretest**	**posttest**	**pretest**	**posttest**
3154 ± 788	2728 ± 482	3342 ± 750	2959 ± 736	3459 ± 724	3131 ± 767	3650 ± 587	3139 ± 376

Note: Descriptive statistics for mean reaction time (in milliseconds) for solving mental arithmetic tasks in the Experimental condition of the Montreal Imaging Stress Task (MIST) by sex and condition; Scores of two participants were excluded from the analysis as outliers; *SD* = standard deviation.
